# HCV Infection in Thalassemia Syndromes and Hemoglobinopathies: New Perspectives

**DOI:** 10.3389/fmolb.2020.00007

**Published:** 2020-01-30

**Authors:** Laura Maffei, Francesco Sorrentino, Patrizia Caprari, Gloria Taliani, Sara Massimi, Roberta Risoluti, Stefano Materazzi

**Affiliations:** ^1^Thalassemia Unit, S. Eugenio Hospital, Rome, Italy; ^2^National Centre for the Control and Evaluation of Medicines, Istituto Superiore di Sanità, Rome, Italy; ^3^Chronic Infectious Diseases Unit, Policlinico Umberto I, Sapienza University of Rome, Rome, Italy; ^4^Department of Chemistry, Sapienza University of Rome, Rome, Italy

**Keywords:** hepatitis C, direct acting antivirals, thalassemia major, sickle cell disease, iron overload, transfusion, liver disease

## Abstract

Hepatitis C virus (HCV) infection is one of the most serious complications of transfusion therapy in the thalassemia and sickle cell disease (SCD) population before 1990; in fact, since 1990 serological tests were made available to detect infection in blood donors. The iron chelation therapy has improved the life expectancy of these patients and, consequently, a decrease in death due to heart disease may be observed, as well as an increase in liver disease due to the iron overload and HCV infection that lead to liver fibrosis, cirrhosis, and hepatocellular carcinoma. Until few years ago, the recommended therapy for HCV treatment consisted of pegylated-interferon alpha plus ribavirin, a therapy with important side effects. This treatment has been severely limited to thalassemic and SCD patients due to the hemolytic anemia induced by ribavirin causing an increase in the number of blood transfusions. The development of highly effective Direct-acting Antiviral Agents toward different viral genotypes has led to a real HCV eradication with negative viremia and sustained viral response between 90 and 98%. At the beginning some indications of Direct-acting Antiviral Agents administration were available for those patients exhibiting advanced cirrhosis or needing liver transplantation over time for the high costs of the new drugs. Recently, all treatment regimens can be used for patients with various HCV genotypes, different stages of liver disease, and comorbidities. The HCV eradication has also led to a marked improvement in the parameters of martial accumulation, demonstrating a synergic action also between the effect of antiviral therapy and iron chelation.

## Introduction

Hepatitis C virus (HCV) infection is one of the most serious complications of transfusion therapy in the thalassemia and Sickle Cell Disease (SCD) population receiving transfusions before 1990; in fact, since the year 1990 serological tests were made available for detect infections in blood donors.

The iron chelation therapy has modified the life expectancy of thalassemia patients; mortality data show a decrease in causes of death due to heart disease and an increase in liver disease (Prati et al., 1998, 2004; Borgna-Pignatti et al., 2004; Agas et al., 2008). Iron overload and HCV infection have been established to be risk factors for thalassemia patients to develop liver fibrosis, cirrhosis, and hepatocellular carcinoma (HCC) (Di Marco et al., 2008; Voskaridou et al., 2012; Borgna-Pignatti et al., 2014; Chung et al., 2015; Moukhadder et al., 2017; Hodroj et al., 2019). Only 15–25% of the infected subjects can eliminate the infection alone. The remaining 75–85% of them make the infection chronic, developing cirrhosis that can be complicated by liver failure and hepatocellular carcinoma. In several studies from different population of thalassemic patients (United States, Italy and Greece) the prevalence of cirrhosis ranged from 10 to 20% and the incidence of HCC in thalassemia patients was progressively increasing (Voskaridou et al., 2012; Borgna-Pignatti et al., 2014; Chung et al., 2015; Moukhadder et al., 2017; Rumi et al., 2018; Hodroj et al., 2019). These data have made an intervention to eradicate HCV infection more urgent.

Regarding Sickle Cell Anemia, about 10–20% of patients have chronic HCV infection. Moreover, due to the need of blood transfusions to manage the sickling crisis and anemia, patients affected by HCV infection frequently are exposed to higher risk of iron overload and hemosiderosis, leading to liver-related morbidity and mortality (Moon et al., 2017). The hepatic involvement ranges in severity from liver dysfunction to liver failure, and occurs as the result of many factors: (a) sickling process (with acute hepatic vaso occlusion, hepatic sickle cell cholestasis, liver ischemia, and reperfusion injury, hemolysis, and cholelithiasis); (b) chronic viral hepatitis; (c) transfusion related hemosiderosis; and (d) autoimmune liver disease (Porter and Garbowski, 2013; Jitraruch et al., 2017; Theocharidou and Suddle, 2019).

## HCV Genotypes

HCV is a heterogeneous virus with at least seven genotypes up to now identified. Each genotype includes multiple subtypes which differ to one another from 31 to 33% over the whole viral genome (Petruzziello et al., 2019). This genetic heterogeneity influences the efficacy of the antiviral therapy as response rate, since it must be specific for different viral strains as concerns type and duration of treatment. Detailed knowledge of HCV genotype has a great clinical relevance since the efficacy of antiviral therapies is greatly influenced by genotypes and subtypes distribution. HCV genotypes are geographically heterogeneous and are characterized by “epidemic subtypes,” such as 1a, 1b, 2a, and 3a in high income countries and the “endemic” strains in restricted areas, such as West Africa, Southern Asia, Central Africa, and Southeastern Asia (Petruzziello et al., 2019). Regardless to Europe, genotype 1 is the most frequent and seems to be equally distributed among European countries (between 50 and 70%) followed by the genotype 3 (between 20 and 29%). About 8.9% of genotype 2 is more frequent in Western Europe, while about 4.9 and 5.8% of genotype 4 may be observed in Central and Western Europe, respectively (Petruzziello et al., 2019).

## The Study Design

In the present study, we report the experience of patients with thalassemia and sickle cell disease treated with DAAs therapy at the Thalassemia Unit of S. Eugenio Hospital of Rome. The study was conducted in accordance with the principles of Good Clinical Practice, the Declaration of Helsinki and all the local regulations. The treatments with DAAs were applied according to criteria defined by national guidelines and were authorized by Italian Medicines Agency. All patients provided their written informed consent to participate in this study.

Effectiveness of the treatment according to the clinical case, was compared to HCV therapy by interferon and DAAs, commonly used to improve the life expectancy of the patients.

## HCV Therapy: Interferon

The treatment of chronic HCV infection began with the administration of interferon (INF) mono therapy, and until a few years ago the recommended therapy available for HCV treatment was pegylated-interferon (PEG-IFN) alpha, plus ribavirin (RBV) with an administration of about 24 weeks (genotypes 2–3) or 48 weeks (genotype 1) (Reddy et al., 2001; Li et al., 2002; Inati et al., 2005; Ricchi et al., 2011; Aminizadeh et al., 2016; Risoluti et al., 2016a,b, 2017, 2018; Catauro et al., 2018). This treatment produced important side effects, such as irritation at the injection site, febrile influenza-like manifestations, mental disorders, thyropathy, neutropenia, and hemolytic anemia. Eradication of the infection did not reach the expected results, due to the presence of subjects who did not respond to therapy or developed recurrences. Infection is assumed eradicated when there is a sustained virological response (SVR), defined as the lack of HCV RNA in serum by a sensitive test performed 24 weeks after completion of antiviral therapy. PEG-IFN plus RBV obtained SVR rates of 25–64% in patients with thalassemia and HCV infection (Inati et al., 2005; Harmatz et al., 2008; Di Marco et al., 2016), and demonstrated an improvement of patient survival due to a reduction in the risk of cirrhosis and hepatocellular carcinoma (Ray and Thomas, 2015). However, this treatment was not well-tolerated by thalassemic and SCD patients and the use has been severely reduced because ribavirin (RBV) proved to cause hemolytic anemia and to increase blood transfusions, in addition to interferon side effects, such as influenza-like symptoms, depression and cytopenias (Materazzi et al., 2014a; Origa et al., 2015; Di Marco et al., 2016; Moon et al., 2017). More recently, the use of Direct-acting Antiviral Agents (DAAs) demonstrated to be suitable in HCV management in patients with thalassemia and sickle cell disease for whom previous regimens gave restrictions (Materazzi et al., 2015, 2017a; Di Marco et al., 2016; Moon et al., 2017; Origa et al., 2017; Premkumar et al., 2017; Mehta et al., 2018).

## HCV Therapy: Direct-Acting Antiviral Agents (DAAS)

In 2011 the development of DAAs gave a breakthrough to the treatment of patients with chronic HCV. The therapy with DAAs was initially performed together with PEG-INF plus RBV for patients with genotype 1 and this led to an increase in SVR to about 70%. The limitation of these treatments consisted of the combination with PEG-INF plus RBV. In 2013, simeprevir and sofosbuvir were introduced. These two drugs were proposed to treat genotype 1 infections with no interferon, and in October 2014 the FDA approved the combination ledipasvir/sofosbuvir to manage HCV genotype 1. Several clinical trials demonstrated a SVR ranging from 94 to 97% after 12 weeks in previously treated and untreated patients, respectively (Afdhal et al., 2014). Subsequently, with the introduction of new types of DAAs, this therapy became available for all HCV genotypes.

At first the indication of DAAs administration were for patients with advanced cirrhosis or needing liver transplantation over time, while recently all regimens have been approved to treat patients with various HCV genotypes, stages of liver disease, and comorbidities. Recent outcomes from clinical trials showed a SVR higher than 95% when LDV/SOF (treatment from 8 to 12 weeks) was used to treat HCV genotypes 1 and 4, including patients with human immunodeficiency virus (HIV) coinfection and cirrhosis (Moon et al., 2017). Several trials have been performed on thalassemia patients, while few studies focused on safety, tolerability and efficacy of LDV/SOF treatment for patients with SCD (Moon et al., 2017). The development in recent years of highly effective and increasingly selective DAAs toward different viral genotypes has led to a real revolution in the HCV eradication for patients with thalassemia and hemoglobinopathies, leading to negative viremia and SVR between 90 and 98% (Materazzi et al., 2014b, 2017b,c; Nagral et al., 2017; Origa et al., 2017; Mehta et al., 2018; Premkumar and Dhiman, 2018; Mangia et al., 2019; Ponti et al., 2019).

A problem on which there has been extensive discussion on the use of DAAs was the cost of such therapies and this has led to give the indication to apply these treatments only to patients with extensive liver disease (F3 or F4). The high costs of the new drugs led to some prescriptive restrictions in the initial phase which unfortunately did not take into account the increase in the neoplastic risk linked to the iron accumulation for the transfusion dependent and hemolytic patients (Chung et al., 2015; Hodroj et al., 2019).

The eradication of the virus determined by the new drugs has also led to a marked improvement in the parameters of martial accumulation, demonstrating a synergic action also between the effect of antiviral therapy and iron chelation (Dharamsi et al., 2017; Nagral et al., 2017; Origa et al., 2017; Premkumar et al., 2017; Mehta et al., 2018; Mangia et al., 2019; Ponti et al., 2019).

## The Experience of the DAAS Therapy of an Italian Thalassemia Center

Thalassemia major, thalassemia intermedia, and sickle cell disease patients with chronic HCV infection and fibrosis stage of F3–F4 were enrolled and then treated with DAAs according to the criteria defined by Italian Medicines Agency and the access to these treatments included a Fibroscan of at least 10 KPa.

Thirty-four patients with a confirmed diagnosis of hemoglobinopathy and HCV infection were treated with DAAs in the period 2015–2018. The characteristics of the patients are described in Table 1. In particular, 28 subjects (16 females and 12 males, mean age 44 ± 7 years) affected by Thalassemia Major, 3 subjects (2 females and 1 males) affected by Thalassemia Intermedia, and 3 subjects (1 female and 2 males) with Sickle Cell Disease (two with HbS/β Thalassemia and one with HbS/HbS). All subjects were transfusion dependent, and because of the iron overload an iron chelation therapy was administered: deferoxamine (DFO) in 29% of patients, deferiprone (DFP) in 13% of patients, deferasirox (DFX) in 25% of patients, DFO + DFP in 23% of patients, and DFO + DFX in 10% of patients. The iron chelation therapies were different and personalized for the patients, the dosages of iron chelators are reported in Table 1.

**Table 1:** Characteristics of the patients treated with DAAs and comparison of serum alanine aminotransferase (ALT), liver iron concentration (LIC), and serum ferritin values between baseline and after treatment at the SVR evaluation.

**Patients, *n***	**34**
**Age, years**	
Mean ± SD	44 ± 7
Range	32–59
**Gender, *n* (%)**	
Male (M)	15 (44.1%)
Female (F)	19 (55.9%)
**Hemoglobinopathy, *n* (%) (M/F)**	
Thalassemia major (TM)	28 (82.4%) 16F/12M
Thalassemia intermedia (TI)	3 (8.8%) 2F/1M
Sickle cell disease (SCD)	3 (8.8%) 1F/2M
**HCV genotype, *n* (%)**	
G1a	3 (8.8%)
G1b	17 (50%)
G2a	5 (14.7%)
G2a/2b	1 (2.9%)
G2a/2c	2 (5.8%)
G3	2 (5.8%)
G4	4 (11.7%)
Liver stiffness, KPa (range)	10.0–34.8
**Iron chelation, mg/Kg/day (days)**	
DFO	30–40 (5–7 days)
DFX	25–35 (7 days)
DFP	60–75 (7 days)
DFO + DFP	25–40 (3–4 days) + 75 (7 days)
DFO + DFX	25–30 (3–6 days) + 20–35 (1–4 days)
**Comorbidities, *n* (%)**	
Heart disease	5 TM (14.7%)
Essential thrombocytopenia	1 TM (2.9%)
Kidney disease	2 SCD (5.9%)
Pulmonary embolism	1 TM (2.9%)
Crioglobulinemia and neuropathy	1TM (2.9%)
Hepato cellular carcinoma	1TM (2.9%)
**Serum ALT, IU/L (mean ± SD, range)**	
Before therapy	44.0 ± 30.1 (9–124)
After therapy *p* = 0.018^*^	24.1 ± 21.8 (8–99)
**LIC, mg Fe/g liver d.w. (mean ± SD, range)**	
Before therapy	1.85 ± 1.22 (0.9–5.7)
After therapy *p* = 0.626^*^	1.66 ± 0.72 (0.9–3.2)
**Serum Ferritin, ng/mL (mean ± SD, range)**	
Before therapy	429 ± 355 (111–1594)
After therapy *p* = 0.150^*^	536 ± 528 (87–2199)

* *Students' t-test for paired data*.

All the patients had previously treated with Interferon to eradicate HCV infection but were not responsive to the therapy. The HCV genotypes were characterized in order to treat the patients with the specific DAAs (Table 1). Among the treated subjects, 50% were genotype 1b (17 subjects), 14.7% were genotype 2a (5 subjects), 11.7% were genotype 4 (4 subjects), 8.8% were genotype 1a (3 subjects), 5.8% were genotype 2a/2c (2 subjects), 5.8% were genotype 3 (2 subjects), and 2.9% were genotype 2a/2b (1 subjects). The liver stiffness, as determined by Fibroscan, showed values between 10.0 and 34.8 KPa.

Some comorbidities were present before treatment in the group of patients. Five thalassemia major subjects suffered with heart disease, and two HbS/β thalassemia cases with kidney disease. Moreover, a case of hepatocellular carcinoma, a cryoglobulinemia with neuropathy, a subject with essential thrombocytopenia, and a case with a pulmonary embolism event were present in thalassemia major patients (Table 1).

The specific DAAs therapies were applied to the patients according to HCV genotype and are reported in Table 2.

**Table 2:** DAAs therapies and virological response after treatment regimens.

**DAAs therapy, *n* (%)**	
Sofosbivir + Ledipasvir	14 (41%)
Sofosbivir + Daclatasvir	4 (12%)
Sofosbuvir + Velpatasvir	9 (26%)
Simeprevir + Sofosbivir	2 (6%)
Sofosbivir	2 (6%)
Elbasvir + Grazoprevir	2 (6%)
Glecaprevir + Pibrentasvir	1 (3%)
**Effectiveness of DAAs therapy**, ***n*** **(%)**	
Sustained Virological Response (SVR)	32/34 (94%)
Not responder with infection relapse	2/34 (6%)

All patients were treated with a cycle (12 weeks) of DAAs, only two Thalassemia major patients were treated for other 12 weeks because they were non-responder to the therapy. No patient required dose reduction or termination of antiviral treatment, and 32/34 patients (94%) reached SVR12 with negative follow up. Two thalassemia major patients with severe comorbidities and treated for 24 weeks, were not responder with infection relapse after 1 month. One of them had hepatocellular carcinoma, and the other had severe dilatation and restrictive cardiomyopathy with liver from stasi. Moreover, a SCD patient died of cholestasis and severe liver failure at 6 months from the end of 12 weeks DAAs therapy without viral recurrence. Already before the treatment the patient had significant hepatic impairment with a high degree of fibrosis (F4), a high fibroscan value 34.8 KPa, liver T2^*^ 13 ms, and normal serum ferritin value (187 ng/mL). To our knowledge the patient did not have a primary sclerosing cholangitis.

All treatment regimens were well-tolerated, and no adverse events were reported. No patient has modified the ongoing iron-chelation treatment and the number of blood transfusions remained unchanged.

The evaluation of some parameters before and after treatment (Table 1) suggested a significant reduction (*p* = 0.018) in alanine aminotransferase levels from 44.0 ± 30.1 IU/L at baseline to 24.1 ± 21.8 IU/L at the SVR evaluation. On the other hand, the liver iron concentration (LIC) resulted decreased after treatment from 1.85 ± 1.22 to 1.66 ± 0.72 mgFe/g liver, but the difference was not significant. Serum ferritin values did not show differences before and after DAAs therapy.

## Discussion

The data currently available in the literature suggest that the eradication of HVC infection using the new DAAs is very close. In addition, the results obtained on the patients with thalassemia and sickle cell disease have given high values of SVR, always higher than 90%. There are still a small percentage of non-responder subjects to DAAs therapy and one of the causes could be that some of them were already very compromised with serious comorbidities when were treated, as in our non-responder subjects with infection relapse after 1 month. Moreover, at first the treatment of patients did not include new types of DAAs currently available. Therefore, now those not responding to the therapy could be treated with other DAAs of more recent generation or with different modalities.

As for the follow-up of patients, the application of DAAs therapies is so recent that there are still not many long-term trial data available to evaluate whether DAAs improve the morbidity and mortality of SVR patients. The patients should be periodically monitored and the follow-up should include the confirm of viral negativity, and the overall assessment of liver disease, particularly in thalassemia and sickle cell disease patients who have an additional risk factor caused by the iron overload. More studies should be performed to evaluate whether the risk of carcinoma is reduced and progressively declined after SVR, but it is still early to make an assessment.

It remains the big problem of the costs of DAAs therapies. These treatment regimens are very expensive, the costs can vary according to the different countries of the world, and this can certainly limit their application, and lengthen the time for a global eradication of HCV.

## Data Availability Statement

The datasets generated for this study are available on request to the corresponding author.

## Ethics Statement

The study was performed in accordance with the principles of Good Clinical Practice, the Declaration of Helsinki, and all the local regulations. The treatments with Direct-acting Antiviral Agents are applied according to criteria defined by national guidelines and are authorized by Italian Medicines Agency (AIFA). All patients provided their written informed consent to participate in this study. The study was approved by Comitato Etico Roma 2, S. Eugenio Hospital, Rome.

## Author Contributions

FS, LM, PC, RR, and SMat contributed to the conception of the study. FS, LM, and GT enrolled the patients, performed the clinical and laboratory evaluation, and management of subjects. FS, LM, PC, SMat, RR, and SMas contributed to the acquisition and evaluation of data for statistics. FS, PC, RR, and SMat wrote the manuscript. All authors have revised and approved the final version of the manuscript.

### Conflict of Interest

The authors declare that the research was conducted in the absence of any commercial or financial relationships that could be construed as a potential conflict of interest.
